# Correlating excessive daytime sleepiness with body mass index, waist circumference, and lipid profile in shift workers

**DOI:** 10.15537/smj.2022.43.11.20220529

**Published:** 2022-11

**Authors:** Diah Kurnia Mirawati, Naomi Ditya Sari, Ervina Arta Jayanti Hutabarat, Yetty Hambarsari, Hanindia Riani Prabaningtyas, Pepi Budianto, Muhammad Hafizhan, Stefanus Erdana Putra

**Affiliations:** *From the Department of Neurology, Faculty of Medicine, Universitas Sebelas Maret, Surakarta, Central Java, Indonesia.*

**Keywords:** excessive daytime sleepiness, Epworth Sleepiness Scale, body mass index, waist circumference, lipid profile

## Abstract

**Objectives:**

To determine the correlation between excessive daytime sleepiness and body mass index, waist circumference, and lipid profile of shift workers at Dr. Moewardi General Hospital, Surakarta.

**Methods:**

This cross-sectional study was carried out at the Dr. Moewardi Hospital, Surakarta, Indonesia between October 2018 and July 2019. The participants were recruited using purposive sampling. Multiple linear regression with backward elimination was performed to identify the odds ratios between Epworth Sleepiness Scale scores, anthropometric measurements, and lipid profiles. A *p*-value of <0.05 indicated statistically significant correlations.

**Results:**

Of the 150 included participants, 127 (84.67%) were women. Statistical analyses revealed odds ratios of 2.38 (95% confidence interval [CI] 1.14-4.89, *p*=0.000) for daytime sleepiness severity and total cholesterol levels, and 2.45 (95% CI 1.36-4.98, *p*=0.020) for daytime sleepiness severity and high-density lipoprotein levels.

**Conclusion:**

Increased total cholesterol and decreased high-density lipoprotein levels increase the risk of excessive daytime sleepiness in shift workers.

**S**leep is essential for human health and wellbeing. The sleep-wake cycle is regulated by a complex circadian rhythm, which is repeated approximately every 24 hours and is influenced by light and hormonal changes. Circadian rhythm disturbances can be caused by intrinsic or extrinsic factors. Intrinsic circadian rhythm disturbances are caused by dysregulation of the internal circadian system due to genetic disorders, resulting in asynchrony between the patient’s sleep/wake cycle and the day/night cycle. In extrinsic circadian rhythm disorders, sleep/wake cycle disturbances are associated with activities such as night shift work or trans-meridian travel.^1^

Shift work is a main cause of circadian rhythm disturbance and sleep deprivation. In the United States, 22 million adults work in shifts, 3.8 million of whom work on regular night shifts. Healthcare workers, including nurses, are required to work on a shift-based schedule to provide 24-hour care for patients. Shift work creates a serious public health problem and negatively affects the quality of life. Shift work can lead to insomnia, sleep insufficiency, psychological stress, increased risk of work and traffic accidents, decreased social interactions, and excessive daytime sleepiness (EDS).^2,3^ Excessive daytime sleepiness is a condition characterized by intense sleepiness with a general lack of energy during the day, resulting in disturbances in work performance, behavior, and quality of life, as well as an increased rates of workplace accidents.^4^

Sleep deprivation also increases the risk of several diseases such as gastrointestinal disorders, cardiovascular disorders, depression, and cancer. Furthermore, it can cause metabolic disorders since sleep plays an important role as a modulator of neuroendocrine function and glucose metabolism. Sleep deprivation is associated with impaired glucose tolerance and impaired regulation of ghrelin and leptin, which regulate appetite. Thus, sleep insufficiency can cause weight gain and obesity.^2,5^ A study of 500 participants in Italy revealed that obesity, elevated cholesterol levels, and elevated triglyceride levels, were significantly increased in shift workers compared to the corresponding levels in regular daytime workers. Another study also found that shift workers had significantly higher body mass indices (BMIs) than those of their colleagues working only during the day. Furthermore, the prevalence of diabetes mellitus and markers of insulin resistance were more frequent in shift workers than in daily workers.^6,7^

This study aimed to understand the effect of shift work by analyzing the correlation among EDS, BMI, waist circumference, and lipid profile of shift workers at Dr. Moewardi General Hospital, Surakarta, Idonesia.

## Methods

This cross-sectional study was carried out at Dr. Moewardi General Hospital, Surakarta, Indonesia. The participants in this study were nurses who worked in wards, emergency rooms, and intensive care units. The inclusion criteria were male or female nurses, aged 20-50 years old, work in a shift schedule for at least one year, and agree to participate in this study. The exclusion criteria were the presence of diabetes mellitus, dyslipidemia, metabolic syndrome, hypertension, heart disease, obstructive sleep apnea, narcolepsy, depression, epilepsy and thyroid disease in the participants, as well as post-menopausal nurses.

Excessive daytime sleepiness was assessed using the Epworth Sleepiness Scale (ESS), which comprises 8 questions that evaluate participant sleepiness in various daily settings. Participants were asked to rate every question on a 4-point scale, from 0 to 3, regarding their chances of experiencing sleepiness in a daily setting. A higher score indicates more severe daytime sleepiness. The duration of shift work was classified as <10 or ≥10 years. Patients were classified into non-EDS (ESS ≤10), mild-to-moderate EDS (11 ≤ ESS ≤ 16), and severe EDS (ESS ≥17) groups.^7,8^

Body height and weight were calculated to determine the BMIs. Body mass indices <18.5 kg/m^2^ are classified as underweight; BMIs of 18.5–24.9 kg/m^2^ are classified as normo-weight; BMIs of 25.0–29.9 kg/m^2^ are classified as overweight; and BMIs ≥30 kg/m^2^ are classified as obese.^9^ We also measured the participants’ waist circumferences and used cutoff values >90 cm for men and >80 cm for women, to be considered abnormal.^10^

Laboratory parameters analyzed in this study were lipid profile, consisting of total cholesterol, low-density lipoprotein (LDL), high-density lipoprotein (HDL), and triglycerides with normal ranges of <200, <130, >40, and <150 mg/dL, respectively.^11^ Laboratory results were obtained by a certified pathologist.

Data distribution was analyzed using the Kolmogorov–Smirnov test. The correlation between shift work durations and ESS scores was determined using Spearman correlation analysis. Correlations between EDS and BMI, waist circumference, and lipid profile were then analyzed using multiple linear regression to determine the odds ratio (ORs). Variables that were statistically significant in the linear regression were then further analyzed with backward elimination to determine the determination coefficient.

### Statistical analysis

Statistical significance was set at a *p*-value of <0.05. All analyses were performed using SPSS version 22 (IBMCorp, Armonk, NY, USA).

## Results

This study was conducted from October, 2018 to July, 2019 at the Dr. Moewardi General Hospital, Surakarta, Indonesia. The total sample size was 154, and 4 participants were excluded from this study. The participants were nurses working in wards, emergency rooms, and intensive care units working in shifts. A total of 150 nurses were assessed for EDS using ESS, BMI, waist circumference, and lipid profiles. The majority of participants were women (84.7%), with a mean age of 32.58±6.003 (range 23-48) years. The Kolmogorov–Smirnov test was used to determine the data distribution. The participants’ demographics, ESS scores, anthropometric data, laboratory data, and data distribution are detailed in Table 1.

**Table 1:** - Participant demographic, Epworth Sleepiness Scale (ESS), anthropometric, laboratory result, and data distribution.

Characteristic	Case		Descriptive			
	n	%	Min	Max	Mean± D	*P*-value
***Gender***						
Male	23	15.3				
Female	127	84.7				
Age			23	48	32.59±6.003	
***Duration of shift work***						
<10 years	68	45.3	1	25	7.98±5.182	
≥10 years	82	54.7				
***ESS Score***						
No EDS	129	86	0	19	4.59±4.154	0.000
EDS	21	14				
***BMI***						
Underweight	11	7.3	16.0	33.8	23.84±3.669	0.003
Normal	93	62.0				
Overweight	33	22.0				
Obesity	13	8.7				
***Waist circumference***						
Normal	63	42	67	116	85.39±10.01	0.033
Abnormal	87	58				
***Total cholesterol***						
Normal	115	76.7	121	299	181.08±31.39	0.011
High	35	23.3				
***LDL***						
Normal	103	68.7	70	212	118.88±29.27	0.001
High	47	31.3				
***HDL***						
Normal	135	90	12	86	52.84±10.73	0.200*
Low	15	10				
***Triglyceride***						
Normal	143	95.3	32	290	88.28±41.58	0.000
High	7	4.7				

*Normal distribution, SD: standard deviation, EDS: excessive daytime sleepiness, BMI: body mass index, LDL: low density lipoprotein, HDL: high density lipoprotein, Min: minimum, Max: maximum

From the data distribution test, HDL was normally distributed with *p*=0.200, while ESS, BMI, waist circumference, total cholesterol, LDL, and triglyceride data were not normally distributed. Spearman’s correlation analysis between the duration of shift work and ESS revealed a correlation coefficient of 0.678 (*p*=0.002). In the linear regression analysis, the total cholesterol and HDL levels were statistically significant, indicating that these parameters were the most important variables correlated with EDS. We identified *p*-values of 0.000 and 0.040 for the total cholesterol and HDL levels, respectively. We then performed further analysis with backward elimination to determine the determination coefficient between EDS, total cholesterol, and HDL. Odds ratio for daytime sleepiness severity and total cholesterol level were 2.38 (95% Confidence Interval [CI] 1.14-4.89, *p*=0.000), and ORs for daytime sleepiness severity and HDL level were 2.45 (95% CI 1.36-4.98, *p*=0.020). The linear regression analysis and backward elimination results are presented in Table 2. The coefficient of determination in the multiple linear regression analysis was 0.583, indicating that 41.7% of daytime sleepiness severity was determined by factors other than total cholesterol and HDL levels.

**Table 2:** - Result of multiple linear regression between Epworth Sleepiness Scale and variables.

Variable	B Coefficient	SE (β)	*P*-value	ORs (95% CI)
***First analysis***				
BMI	0.140	0.012	0.165	2.53 (1.29-4.97)
Waist circumference	0.085	0.005	0.423	2.19 (1.11-4.34)
Total cholesterol	0.833	0.004	0.000	2.12 (1.08-4.18)
HDL	-0.641	0.001	0.040	2.23 (1.19-4.23)
LDL	0.508	0.002	0.612	1.87 (1.01-4.10)
Triglyceride	0.098	0.069	0.286	2.02 (1.09-4.31)
***Second analysis***				
Total cholesterol	0.791	0.002	0.000	2.38 (1.14-4.89)
HDL	-0.116	0.001	0.020	2.45 (1.36-4.98)

*Statistically significant. SE: standard error, CI: confidence interval, ORs: odds ratio BMI: body mass index, LDL: low density lipoprotein, HDL: high density lipoprotein

## Discussion

A survey by the Ministry of Health, Republic of Indonesia, in 2018 revealed that 71% of nurses are female,which was represented in our study population.^12^ The present study identified a 14% prevalence of EDS with an average ESS score of 4.59±4.15. The prevalence of EDS in the young adult population varies greatly between studies; however, approximately 20% of the young adult population reportedly suffers from EDS.^4^

The prevalence of overweight in this study was 22% and obesity was 8.7%, similar to the prevalence of overweight in the general population in Indonesia, based on Indonesian National Health Research, which was 19.7% in men and 32.9% in women.^13^ The prevalence of overweight in this study was lower that reported by Gadallah et al^14^ who evaluated the relationship between shift work and lipid profiles in 86 nurses at the Egyptian University Hospital, identifying a prevalence of 41.9%. Furthermore, the prevalence of an abnormal waist circumference was 58% ranging from to 67–116 cm. Approximately 42% of participants had a normal waist circumference, similar to the values reported by Marqueze et al^15^ evaluating the effect of shift work on cardiovascular risk factors in 30 truck drivers in Brazil, where the prevalence of an abnormal waist circumference was 42.3%.

In this study, 2 anthropometric measurements, BMI and waist circumference, were used to assess the proportion of body fat. This study revealed that the prevalence of an abnormal waist circumference was almost twice as high as the prevalence of overweight BMI. This is because BMI, unlike waist circumference, does not reflect the body fat distribution. The American Heart Association, National Heart, Lung, and Blood Institute, and the International Diabetes Federation recommend the use of waist circumference as a screening tool for diagnosing metabolic syndrome. Waist circumference can also serve as an indicator of central obesity and a better predictor of cardiovascular diseases compared to BMI.^16^ However, the WHO still recommends measuring both BMI and waist circumference to assess metabolic syndrome.^9^

In this study, 23.3% of the participants had total cholesterol levels >200 mg/dL. The total cholesterol level in our study ranged from 121-299 mg/dL with an average of 185.08±31.4 mg/dL, which was higher than the values reported by other studies, which reported high total cholesterol prevalence values of approximately 21.7%.^15^ Moreover, 31.3% of the present study’s participants had high LDL levels, which conformed with Marqueze’s et al^15^ study, who found high LDL levels in approximately 34.8% shift worker. A study carried out at a teaching hospital in Egypt, attempting to understand the relationship between shift work and lipid profile in 86 nurses, found high LDL levels in 43% of participants. High LDL levels indicate high tissue cholesterol transport, which increases the risk of cardiovascular and cerebrovascular diseases.^14^

Additionally, 10% of the present study’s participants had low HDL levels. The mean HDL level in our study was 52.84±10.73 (range 12-86) mg/dL. This percentage is much lower than that reported by Marqueze et al^15^ on shift workers, who reported a low prevalence of HDL (approximately 34.8%). Similarly, another study on the relationship between shift work and lipid profile in 86 nurses found that 30.2% of the participants had low HDL.^14^ High-density lipoprotein is a lipoprotein that transports excess lipids from the tissues to the liver for excretion and reuse. Low HDL levels increase the risk of heart disease, hypertension, and cerebrovascular diseases. The risk of coronary heart disease and hypertension increases by 2 to 3% for every 1.0 mg/dL decrease in HDL cholesterol levels.^17^ High triglyceride levels were found in 4.7% of participants. The average triglyceride was 52.84±10.73 mg/dL, ranging from 32 to 290 mg/dL. This prevalence is lower than the high triglyceride prevalence of 30.4% in shift workers reported by Marqueze ey al.^15^ A higher prevalence of high triglyceride levels in shift workers was reported by Gadallah et al^14^ in Egypt (59.3%) and Biggi et al^18^ in Italy (41.5%).

Spearman correlation analysis revealed a correlation coefficient of 0.678 (*p*=0.002), indicating a strong correlation between shift work duration and ESS. Sleep is vital for proper functioning in humans. Many shift workers have irregular sleep schedules, which have many consequences such as impacting working and learning performance. The sleep-wake cycle is regulated by the circadian rhythm, a 24-hour cycle that is part of the human internal clock. Light exposure during the day triggers the central clock in the suprachiasmatic nuclei of the hypothalamus and suppresses the pineal gland to reduce melatonin secretion. At night, shift workers are exposed to light, which disrupts their circadian rhythm. Unmet sleep needs lead to sleepiness after night shifts. Workers performing long shifts, which are greater than 12 hours, tend to experience greater sleepiness and performance impairment.^19,20^

Our multiple regression analysis revealed that the total cholesterol level was statistically significant with *p*-value of 0.000 and HDL level was statistically significant with *p*-value of 0.040. This finding indicates that these parameters are the most important variables correlated with the EDS. We further analyzed these data with multiple linear regression to determine the ORs. The ORs for daytime sleepiness severity and total cholesterol levels were 2.38 (95% CI 1.14-4.89, *p*=0.000), indicating that increased total cholesterol level is associated with 2.38-fold increased risk of daytime sleepiness. Furthermore, the ORs for daytime sleepiness severity and HDL level were 2.45 (95% CI 1.36-4.98, *p*=0.020) indicating that decreased HDL levels are associated with 2.45–fold increased risk of daytime sleepiness. Increasing levels of total cholesterol, LDL, and triglycerides, and decreased HDL levels in EDS are caused by decreasing energy expenditure due to alteration of sympathetic nerves, resulting in a decrease in leptin and changes in diet due to increasing ghrelin levels. Two possible mechanisms may cause an increase in the lipid profile in EDS. The first mechanism is the disruption of the circadian rhythm through impaired melatonin production due to sleep insufficiency. Disruption of melatonin production causes Circadian Locomotor Output Cycles Kaput protein deficiency, resulting in increased absorption of carbohydrates and fats in the intestines throughout the day, and triggers fat accumulation in the liver.^21,22^ The second mechanism is a change in diet. Research has revealed that night shift workers have an appetite for foods that are high in simple carbohydrates and saturated fats. Moreover, shift workers tend to have low fiber and high saccharose intake, which may affect serum lipoprotein levels.^23^

The coefficient of determination in the multiple linear regression analysis was 0.583, indicating that 41.7% of daytime sleepiness severity was determined by factors other than total cholesterol and HDL levels. Several factors, such as sleep deprivation, insomnia, and female sex are more prone to EDS. Sleep problems such as talking, waking up, and teeth grinding also increase daytime sleepiness.^24,25^

These findings underline the risk of dyslipidemia in nurses working on shift schedules. Shift schedules also pose risks to patient safety owing to decreased focus and executive function. To mitigate these risks, hospital administrators could arrange nurses’ shifts to take at least 2 days off after 2 consecutive night shifts, before starting a day shift. Chang et al^26^ determined that one day off after 2 night shifts is insufficient to maintain visual attention performance and executive function ability. Another possible measure is to assign more nurses to night shifts, to enable nurses to take a nap during their shift. A recent study revealed that a quick, 30-minute nap during a night shift can result in less fatigue, less sleepiness, reduced medical errors, and increased psychomotor performance.^27-29^

Due to the high comorbidity that causes EDS in shift workers, other factors that can worsen this condition must be considered. Further research should explore and analyze other influential factors, such as daily activities or stressful conditions that may cause EDS in shift workers.

### Study limitations

This study had some biases that might not have been excluded. We used a self-administered questionnaire, which could have been affected by recall bias. Moreover, the sample size was relatively small; therefore, analyses based on gender could not be performed. Further research following a longitudinal cohort design may be able to include more samples because the number of shift workers is large.

In conclusion, increased total cholesterol and decreased HDL levels were associated with an increased risk of EDS in shift workers at the Dr. Moewardi Hospital Surakarta.
